# Disparidades Étnicas na Longevidade dos Medalhistas Olímpicos Brasileiros

**DOI:** 10.36660/abc.20240082

**Published:** 2024-04-09

**Authors:** Filipe Ferrari, Arthur Proença Rossi

**Affiliations:** 1 Universidade Federal do Rio Grande do Sul Hospital de Clínicas de Porto Alegre Porto Alegre RS Brasil Programa de Pós-Graduação em Cardiologia e Ciências Cardiovasculares – Universidade Federal do Rio Grande do Sul, Hospital de Clínicas de Porto Alegre, Porto Alegre, RS – Brasil; 2 Universidade Federal do Rio Grande do Sul Hospital de Clínicas de Porto Alegre Porto Alegre RS Brasil Grupo de Pesquisa em Cardiologia do Exercício (CardioEx) – Universidade Federal do Rio Grande do Sul, Hospital de Clínicas de Porto Alegre, Porto Alegre, RS – Brasil

**Keywords:** Atletas, Esportes, Grupos Étnicos, Longevidade

A inatividade física é um contribuinte bem documentado para doenças cardiovasculares, diabetes e certos tipos de câncer, sendo responsável por aproximadamente 30% das doenças cardiovasculares, 27% da diabetes e 21-25% dos cânceres da mama e do cólon.^1^ O impacto e os custos da inatividade física associados à saúde pública são substanciais, atingindo INT$ 53,8 bilhões em 2013.^2^

Em termos de longevidade, a prática de atividade física tem sido associada a uma redução dos principais fatores de risco de mortalidade, incluindo hipertensão, diabetes, doença coronária, acidente vascular cerebral e câncer. Estudos indicam uma clara relação dose-resposta entre atividade física moderada a vigorosa e mortalidade por todas as causas, sendo a redução do risco por unidade de tempo maior para atividades mais vigorosas.^3^ Indivíduos fisicamente ativos experimentam uma redução de até 35% na mortalidade por todas as causas em comparação com os seus homólogos inativos, aumentando a esperança de vida em até 7 anos.^3^

Apesar dos benefícios bem estabelecidos da atividade física, os estudos que examinam o impacto de fatores étnicos, sociais e demográficos na mortalidade, especialmente entre atletas, e ainda mais em atletas de alto rendimento ao longo das décadas, são limitados. A recolecção de dados sobre a prevalência ou complicações de doenças por raça, etnia e outras identidades é frequentemente realizada em investigação médica, por departamentos de saúde pública e por sistemas de saúde individuais, mas não é muito comum em estudos de medicina desportiva. Estes dados são cruciais para compreender as disparidades na saúde e os seus mediadores. Quando devidamente analisados, os dados sobre diferenças a nível de grupo podem ser utilizados para impulsionar intervenções direcionadas a grupos para eliminar disparidades.^4^

Em estudo recente dos ABC Cardiol, Braga et al.^5^ investigaram a sobrevivência pós-medalha de medalhistas olímpicos brasileiros de 1920 a 1992, categorizando os atletas em brancos e não-brancos com base na determinação estruturada da etnia. As descobertas revelaram disparidades significativas nas taxas de mortalidade, com atletas não brancos enfrentando um risco significativamente elevado de mortalidade em comparação com atletas brancos. Atletas brancos demonstraram uma expectativa de vida seis anos a mais do que seus colegas não brancos após ganharem uma medalha. Como resultado, entre 123 atletas (73,9% brancos), a idade média em que conquistaram medalhas foi de 25,03 ± 4,8 anos. Durante o estudo, 18,7% dos atletas brancos e 37,5% dos atletas não brancos morreram (p = 0,031). Os atletas brancos tiveram média de idade ao óbito de 75,10 ± 18,01 anos, enquanto os não brancos tiveram média de idade de 67,13 ± 14,90 anos (p = 0,109). O tempo médio de sobrevivência restrito (TMSR), que é uma nova medida alternativa em análises de sobrevivência definida como a área sob a curva de sobrevivência até um ponto de tempo específico,^6^ para atletas brancos foi de 51,59 (intervalo de confiança [IC] de 95%, 49,79 a 53,39 anos), e para atletas não brancos, foi de 45,03 (IC95%, 41,31 a 48,74 anos), resultando em um ΔTMSR de 6,56 (95% IC, 2,43 a 10,70; p = 0,0018).

Segundo o estudo, os atletas olímpicos brasileiros brancos tinham uma expectativa de vida seis anos a mais do que os atletas não brancos após a conquista da medalha. Apesar disso, os dados do estudo demonstram uma discrepância significativa na mortalidade entre os grupos étnicos, mesmo dentro de uma população de atletas de alto rendimento. A literatura sugere que a atividade aeróbica e de resistência regular em idosos demonstrou melhorar a função musculoesquelética e a mobilidade, além de manter a capacidade sensorial para um envelhecimento saudável.^7 - 9^ Isso pode ter um grande impacto na expectativa de vida. Porém, como limitação, não podemos determinar se os atletas do estudo ainda estavam expostos à atividade física regular. Esta poderia ser uma análise que vale a pena considerar, especialmente porque indivíduos brancos e não brancos podem ter acesso diferente a atividades físicas regulares, mesmo dentro de um grupo de atletas vencedores de medalhas olímpicas.

Embora o estudo contribua com informações valiosas sobre as disparidades de saúde entre atletas de elite, não é isento de limitações. A categorização baseada em fotografias introduz possíveis erros de classificação e o tamanho da amostra permanece limitado. Além disso, alguns pontos seriam interessantes de serem discutidos, como as comorbidades subjacentes e os esportes praticados antes e depois da medalha. Além disso, as causas específicas das mortes dos atletas enriqueceriam este estudo, permitindo-nos compreender melhor os dados apresentados. Alcançar uma inclusão abrangente de todos os atletas olímpicos brasileiros é um desafio constante, e a falta de informações sobre as causas específicas de morte e uma comparação direta com a expectativa de vida da população em geral são limitações dignas de nota.

Em conclusão, este estudo sublinha a importância de considerar a raça e a etnia na avaliação da saúde e da esperança de vida dos atletas de elite. As disparidades observadas nas taxas de mortalidade entre os atletas olímpicos brasileiros com base na etnia revelam uma área potencial para intervenções direcionadas para abordar as desigualdades em saúde, mesmo no âmbito dos esportes de alto rendimento.
