# Supplementary material for: Disparidades Étnicas na Longevidade dos Medalhistas Olímpicos Brasileiros
Source: Arq Bras Cardiol. 2024 Apr 9;121(2):e20240082. [Article in Portuguese] doi: 10.36660/abc.20240082 (PMC11081096; doi:10.36660/abc.20240082)
Supplement: Translation

# Translation: Ethnic Disparities in the Longevity of Brazilian Olympic Medalists

**Authors:** Filipe Ferrari, Arthur Proença Rossi

**Affiliations:** 1 Porto Alegre RS Brazil Programa de Pós-Graduação em Cardiologia e Ciências Cardiovasculares – Universidade Federal do Rio Grande do Sul, Hospital de Clínicas de Porto Alegre, Porto Alegre, RS – Brazil; 2 Porto Alegre RS Brazil Grupo de Pesquisa em Cardiologia do Exercício (CardioEx) – Universidade Federal do Rio Grande do Sul, Hospital de Clínicas de Porto Alegre, Porto Alegre, RS – Brazil

**Keywords:** Atletes, Sports, Ethnic Groups, Longevity

Physical inactivity is a well-documented contributor to cardiovascular diseases, diabetes, and certain cancers, accounting for approximately 30% of cardiovascular disease, 27% of diabetes, and 21–25% of breast and colon cancer.^1^The impact and costs associated with public health are substantial, amounting to a total direct health cost due to physical inactivity related to the main non-communicable diseases, which reached INT$ 53.8 billion in 2013.^2^

In terms of longevity, engaging in physical activity has been linked to a reduction in major mortality risk factors, including hypertension, diabetes, coronary heart disease, stroke, and cancer. Studies indicate a clear dose-response relationship between moderate-to-vigorous physical activity and all-cause mortality, with risk reduction per unit of time being greater for more vigorous activity.^3^Physically active individuals experience up to a 35% decrease in all-cause mortality compared to their inactive counterparts, increasing life expectancy by up to 7 years.^3^

Despite the well-established benefits of physical activity, studies examining the impact of ethnic, social, and demographic factors on mortality, especially among athletes, and further on high-performance athletes over the decades, are limited. The collection of data on disease prevalence or complications by race, ethnicity, and other identities is often performed in medical research, by public health departments, and by individual health systems, but it is not very common in sports medicine studies. These data are crucial to understanding health disparities and their mediators. When properly analyzed, data on group-level differences can be used to drive group-targeted interventions to eliminate disparities.^4^

In a recent study from ABC Cardiol, Braga et al.^5^investigated the post-medal survival of Brazilian Olympic medalists from 1920 to 1992, categorizing athletes into white and non-white based on structured ethnicity determination. The findings revealed significant disparities in mortality rates, with non-white athletes facing a significantly elevated risk of mortality compared to white athletes. White athletes demonstrated a life expectancy of six years longer than their non-white counterparts after winning a medal. As a result, among 123 athletes (73.9% white), the mean age at which they achieved medals was 25.03 ± 4.8 years. During the study, 18.7% of white athletes and 37.5% of non-white athletes died (p = 0.031). White athletes had a mean age at death of 75.10 ± 18.01 years, while non-whites had a mean age of 67.13 ± 14.90 years (p = 0.109). The Restricted mean survival time (RMST), which is a novel alternative measure in survival analyses defined as the area under the survival curve up to a specific time point,^6^for white athletes was 51.59 (95% confidence interval [CI], 49.79 to 53.39 years), and for non-white athletes, it was 45.03 (95%CI, 41.31 to 48.74 years), resulting in a ΔRMST of 6.56 (95%CI, 2.43 to 10.70; p = 0.0018).

According to the study, white Brazilian Olympic athletes had a life expectancy of six years longer than non-white athletes after winning the medal. Despite this, the data from the study demonstrates a significant discrepancy in mortality between ethnic groups even within a population of high-performance athletes. Literature suggests that regular aerobic and resistance activity in older adults has been shown to improve musculoskeletal function, and mobility, and maintain sensory capacity for healthy aging.^7 - 9^This may have a great impact on life expectancy. However, as a limitation, we cannot determine if the athletes in the study were still exposed to regular physical activity. This could be an analysis worth considering, especially since white and non-white individuals may have different access to regular physical activities, even within a group of Olympic medal-winning athletes.

While the study contributes valuable insights into health disparities among elite athletes, it is not without limitations. Categorization based on photographs introduces possible classification errors, and the sample size remains limited. Furthermore, some points would be interesting to discuss, such as the underlying comorbidities, and the sports practiced before and after the medal. Furthermore, the specific causes of the athletes’ deaths would enrich this study, allowing us to better understand the data presented. Achieving a comprehensive inclusion of all Brazilian Olympic athletes is an ongoing challenge, and the lack of information about the specific causes of death and a direct comparison with the general population's life expectancy are noteworthy limitations.

In conclusion, this study underscores the importance of considering race and ethnicity in assessing the health and life expectancy of elite athletes. The observed disparities in mortality rates among Brazilian Olympic athletes based on ethnicity reveal a potential area for targeted interventions to address health inequalities, even in the realm of high-performance sports.
